# Chronic circadian misalignment is a risk factor for hair growth impairment

**DOI:** 10.1016/j.isci.2024.110974

**Published:** 2024-09-18

**Authors:** Yoshiki Miyawaki, Atsuhiro Nishida, Keisuke Fukushima, Aoi Matsumoto, Teruki Hamano, Yukiya Mori, Mamoru Nagano, Isao T. Tokuda, Yasufumi Shigeyoshi, Koichi Node, Makoto Akashi

**Affiliations:** 1 The Research Institute for Time Studies, Yamaguchi University, 1677-1 Yoshida, Yamaguchi, Yamaguchi, Japan; 2 Department of Anatomy and Neurobiology, Kindai University, 377-2 Ohno-Higashi, Osaka-Sayama, Osaka, Japan; 3 Department of Mechanical Engineering, Ritsumeikan University, 1-1-1 Nojihigashi, Kusatsu, Shiga, Japan; 4 Department of Cardiovascular Medicine, Saga University, 5-1-1 Nabeshima, Saga, Saga, Japan

**Keywords:** Surface anatomy, Systems biology

## Abstract

To identify environmental factors that accelerate hair loss, we focused on modern artificial and aberrant light environments which cause circadian dysfunction. We examined the effect of aberrant light environments on hair growth by exposing mice to repetitive light-dark reversal at three-day intervals, inducing chronic circadian misalignment. Dorsal hair-sheared male mice showed impaired hair growth under this light condition. In addition, synchronization of hair growth cycling by dorsal depilation in male and female mice revealed that this light condition caused a decrease in hair growth rate during anagen. Furthermore, a decrease in hair growth rate was confirmed in male mice by *ex vivo* culture of whisker hair follicles classified as in anagen. These lines of evidence indicate that artificial and aberrant light environments or chronic circadian misalignment cause impaired hair growth due to a decrease in hair growth rate during anagen and are therefore a potential risk factor for hair loss.

## Highlights

• Chronic circadian misalignment caused an impairment in dorsal hair growth in mice
• This impairment was attributable to a decrease in hair growth rate during anagen
• This decrease was also detected by *ex vivo* culture of whisker hair follicles

## Graphical abstract

**Figure:**
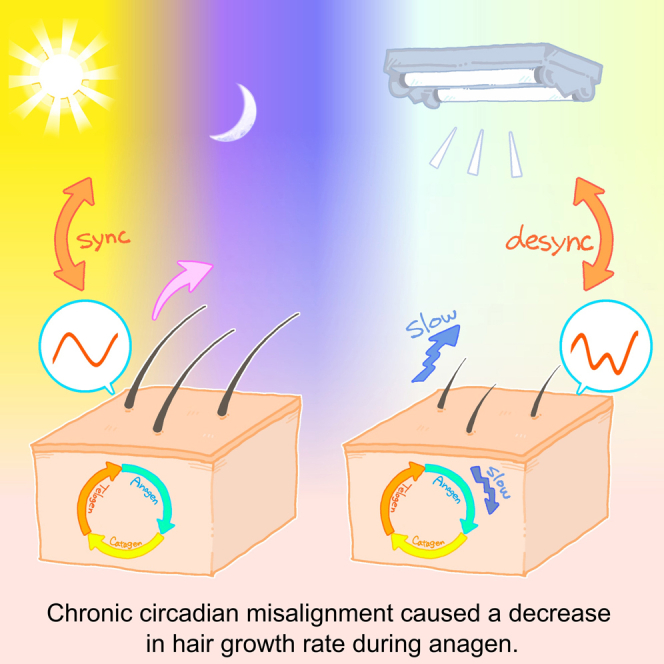

## Introduction

In almost all organisms, the circadian clock, an internal oscillator, drives circadian rhythms,^1^ which are synchronized to the natural 24 h light/dark cycle via the sensing of environmental cues.^2^ The clock consists of interconnected transcription and translation feedback loops.^3^ These molecular loops then generate circadian expression of a wide range of genes, which in turn leads to circadian rhythms in diverse biological processes.^4^ This genome-wide orchestration of gene expression allows the organism to adapt to the earth’s rotation. The circadian oscillator is cell-autonomous and exists in most types of cells, even cultured immortalized cells.^5^^,^^6^ Numerous peripheral circadian clocks throughout the body are governed by humoral and neuronal signals produced by a central clock, the suprachiasmatic nucleus, which is a defined set of cell clusters in the anteroventral hypothalamus.^7^

Circadian clock-driven internal and socially enforced rhythms are often misaligned in modern people. A European study using a chronotype questionnaire indicated that one-third of the population suffers from a social jet lag of 2 h or more.^8^ In particular, night workers, whose daily habitual schedules, including waking, sleeping and meal times, change dramatically in accordance with their working schedule, suffer from chronic misalignment between circadian and social rhythms. Approximately 20% of workers in developed countries are involved in night work.^9^^,^^10^ Night workers have been used in epidemiological studies conducted to reveal human health problems caused by circadian dysfunction. Many of these studies have strongly suggested that night work-induced chronic circadian misalignment increases the risk of a diverse range of health problems, including sleep disorders, metabolic syndrome, cardiovascular diseases and cancer.^11^^,^^12^^,^^13^^,^^14^

Although we are unaware of any epidemiological study of the relation between night work and hair loss, animal studies have clearly demonstrated a negative impact of clock gene dysfunction on hair growth. Among these, all 30-week-old wild-type mice tested showed complete hair regrowth after shaving in one month, whereas only 20% of *Bmal1* knockout mice exhibited partial regrowth after 3 months.^15^ This defect was age-related since 10-week-old knockout mice displayed normal hair regrowth. Consistent with these findings, analysis of mice lacking functional *Clock* and *Bmal1* revealed a delay in anagen progression and a decreased number of mitotic cells in the secondary hair germ.^16^ Furthermore, an *ex vivo* study suggested the involvement of clock proteins in human hair growth: Knockdown of either BMAL1 or Period1 protein in human hair follicles significantly prolonged anagen.^17^ Importantly, however, a recent study indicated that many phenotypes in conventional clock gene knockout mice, including hair growth defect, reflect the loss of properties of clock protein that are independent of its role in the circadian clock.^18^ Specifically, conditional *Bmal1* knockout mice lacking the BMAL1 protein during adult life show normal or accelerated hair regrowth after shaving, in comparison with wild-type mice. Related to this, other studies have demonstrated that whereas initiation of the first anagen is clearly delayed in whole-body *Bmal1* knockout mice,^16^ epithelium-specific knockout of *Bmal1* does not cause this delay,^19^ indicating that this impairment is attributable not to circadian dysfunction in the epithelium but to systemic and/or non-epithelial cell-mediated effects. The drawing of conclusions on the effect of circadian dysfunction on hair growth may therefore require reevaluation with different experimental approaches. In terms of translational significance for public health, knockout and knockdown approaches are not preferable because the circadian dysfunction they cause is fundamentally different from that in the human real world.

Here, we reevaluated the effect of circadian dysfunction on mouse hair growth under chronic misalignment between clock-driven internal and environmental rhythms. More specifically, we eschewed knockout and knockdown approaches, and instead used an experimental model of repetitive reversal of light-dark (LD) cycles to examine the effect of circadian misalignment on mouse hair growth. We examined hair growth performance not only *in vivo* but also in an *ex vivo* culture of hair follicles. Given the strong effect of sex hormones on hair growth and loss, we also focused on sex differences in the effect of circadian misalignment on hair growth.

## Results

### Effects of repetitive LD reversal on dorsal hair growth *in vivo*

**Figure 1:**
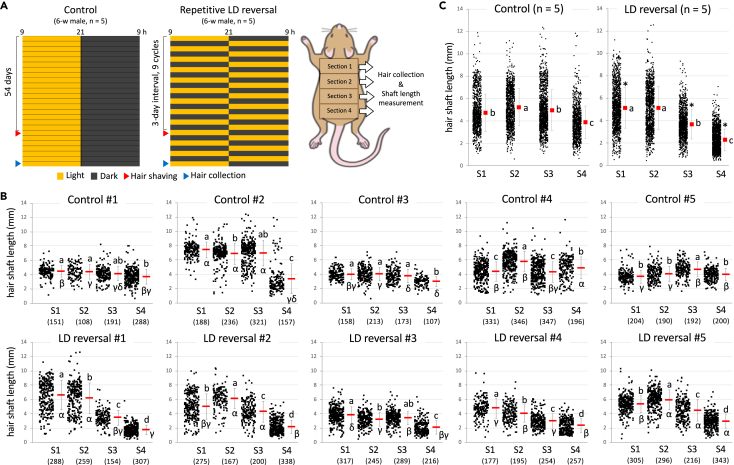
Effects of repetitive LD reversal on dorsal hair growth *in vivo* (A, left and middle) Schematic schedules of the dorsal hair growth assay. After a regular 12:12 LD cycle for habituation, 10 six-week-old male wild-type C57BL6J mice were housed singly and half were exposed to a repetitive LD reversal cycle at three-day intervals for a total of 54 days. Their dorsal hair shafts were neatly sheared short at the day indicated by red triangles. (A, right) Dorsal area was divided into four equal sections, and hair shafts were randomly collected from each section 18 days after shearing, indicated by blue triangles in the schedules. (B) The length of each plucked hair shaft was measured based on digital images. Each dot represents a single dorsal hair shaft. S1 to S4 represent dorsal sections and numbers in parentheses are the total number of hair shafts examined. Horizontal red and error bars indicate the average and standard deviation of hair shaft length, respectively. Horizontal bars labeled with different letters are significantly different (two-way ANOVA with post hoc Tukey’s test, *p* < 0.01). Alphabet, inter-section comparison in each animal. Symbol, inter-mouse comparison in each section. (C) Hair shaft length data from all 5 mice were combined and integrated in each experimental group. A two-way ANOVA with post hoc Tukey’s test was performed. Different alphabet letters represent significant differences among four dorsal sections in each LD condition (*p* < 0.01). Asterisks represent a significant difference between LD conditions in each section (*p* < 0.01).

Mice carrying a dysfunctional clock gene show drastic phenotypes in hair growth cycle, as described above. The clinical significance of these phenotypes is limited, however, because they vary depending on gene disruption method and because the circadian dysfunction in these mice fundamentally differs from that in the human real world. To reevaluate the effect of circadian dysfunction on the hair growth cycle without using genetically engineered mice, we exposed male wild-type mice to repetitive reversal of LD cycle at three-day intervals for a total of 54 days (Figure 1A, experimental schedules). In other words, we examined the effect of forced and chronic circadian misalignment similar to that produced by rotating shift work on hair growth cycle. A number of previous studies have used similar irregular LD schedules as a shift work model.^20^^,^^21^^,^^22^^,^^23^^,^^24^ Here, we performed a three-day interval light and dark reversal because similar schedules are actually used in some real shift work. In consideration of isolation stress, the exposure period was limited to around two months by reference to an isolation period usually applied to monitoring circadian locomotor activity. Actograms of mice exposed to repetitive LD reversal show that circadian rhythms of locomotor activity were incompletely synchronized to LD cycles (Figure S1A). Body weight gain showed no statistically significant difference between the control and LD reversal groups, indicating that chronic LD reversal resulted in no severe health problems (Figure S1B). In addition to locomotor activity, we confirmed that circadian rhythms of core body temperature were also unusual and desynchronized from LD cycles under a repetitive LD-reversal condition (Figure S2).

Grooming clippers were used to neatly shear dorsal hair shafts to an average length of approximately 0.7 mm immediately after repetitive LD reversal for 54 days. 18 days later, the dorsal area from the root of the neck to the base of the tail was divided into four equal sections, from which hair shafts were randomly collected using tweezers and the length of each hair shaft was measured under a stereo-microscope (Figure 1A, right). Control mice showed similar hair growth among all dorsal sections, with a single exception (Control #2) (Figure 1B, top graphs). In contrast, more caudal sections showed more delayed hair growth in all mice exposed to repetitive LD reversal (Figure 1B, bottom graphs). All hair shaft length data from the five mice in both experimental groups are integrated in Figure 1C, which shows a significant delay in hair growth in sections S3 and S4 of mice exposed to repetitive LD reversal.

### Effects of repetitive LD reversal on hair regrowth following depilation-induced synchronization of hair growth cycling

**Figure 2:**
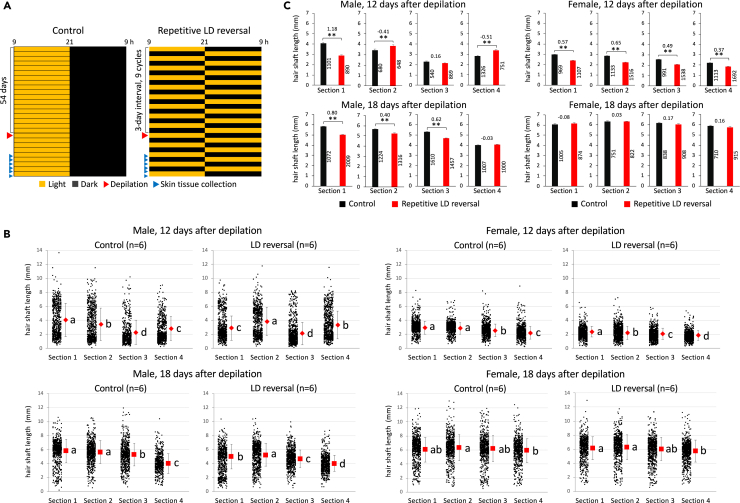
Effects of repetitive LD reversal on hair regrowth following depilation-induced synchronization of hair growth cycling (A) Schematic schedules of the dorsal hair regrowth assay under a depilation condition. Six-week-old male and female wild-type C57BL6J mice were housed singly. After a regular 12:12 LD cycle for habituation, half of the mice were exposed to a repetitive LD reversal cycle at three-day intervals for a total of 54 days. All mice were anesthetized following exposure to the light environments, and their dorsal hair shafts were completely removed by waxing at the day indicated by red triangles. (B) As in Figure 1, dorsal area was divided into four equal sections, from which hair shafts were randomly collected 12 and 18 days after waxing. The length of each plucked hair shaft was measured based on digital images. Each dot represents a single dorsal hair shaft. S1 to S4 represent dorsal sections. Horizontal red and error bars indicate the average and standard deviation of hair shaft length, respectively. (C) To facilitate visual comparison between the control and LD-reversal groups in each section, the data in (b) are shown as bar graphs plotting the average hair shaft length in a section to section manner. Numbers (mm) above columns are the difference in the average hair shaft length (control minus LD reversal). Numbers beside columns are the total number of hair shafts examined. (B and C) two-way ANOVA with post hoc Tukey’s test (factor1, LD condition; factor2, section) was performed for each sex on each day. In (b), squares labeled with different alphabet letters represent a significant difference among four sections in each LD condition (*p* < 0.01). In (c), asterisks represent a significant difference between LD conditions in each section (∗*p* < 0.05, ∗∗*p* < 0.01).

Hair growth cycle phases in the back are likely different from the nuchal to caudal area in young mice.^25^ It is therefore unclear whether any difference in anatomical area or hair growth cycle phase contributed to the delay in hair growth in the caudal dorsal area of mice exposed to repetitive LD reversal. To address this issue, following repetitive LD reversal for 54 days, hair regrowth was examined after depilation of dorsal hair shafts by waxing (Figure 2A), as growth cycle is tightly synchronized by this procedure.^25^ Given previous reports that hair physiology and pathology are affected by the difference in either or both plasma concentrations and ratio of sex hormones,^26^^,^^27^^,^^28^ we examined sex-dependent responses to circadian misalignment using female in addition to male mice. Dot plots showing all individual shaft lengths indicate that 12 days after hair depilation, the data from male mice show a very large variation among not only dorsal sections but also hair shafts in each section in comparison with female mice, and that 18 days after depilation, female hair shafts were longer than male ones in all dorsal sections, regardless of LD conditions, indicating that female shafts grew and matured faster than male ones (Figure 2B). 12 days after hair depilation, female mice showed a statistically significant delay in hair regrowth in all dorsal sections compared to control mice (Figure 2C, top bar graphs). In male mice, however, the results were inconsistent among dorsal sections. In contrast, 18 days after hair depilation, a statistically significant delay in dorsal hair regrowth was detected in male but not female mice exposed to repetitive LD reversal (Figure 2C, bottom bar graphs). A patch with hair growth cycle phase desynchronized from other dorsal areas frequently appears in male mice receiving hair depilation.^25^ A statistical test using data without hair shafts collected from sections including any such patch showed similar results (Figure S3).

### Histological investigation of temporal changes in the percentage of hair growth cycle stages in male and female mice

**Figure 3:**
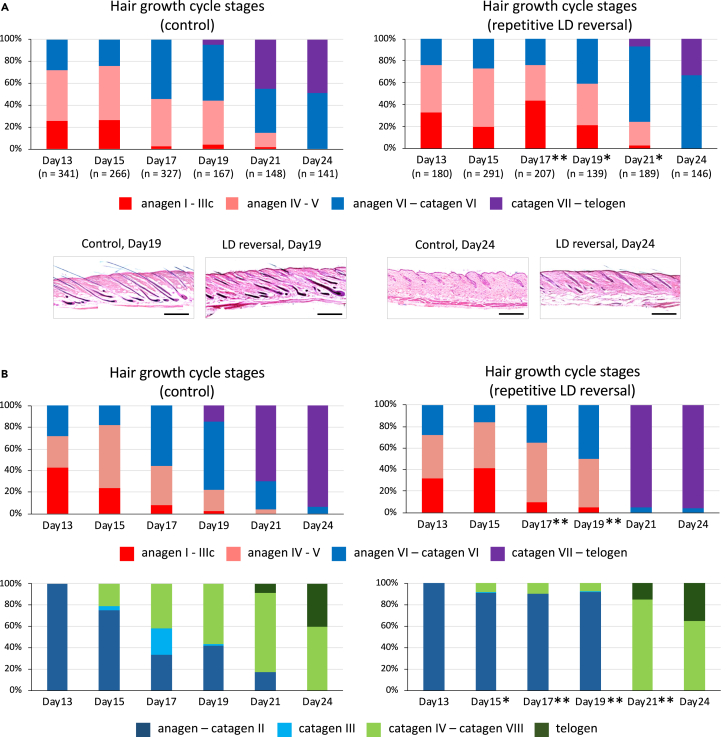
Histological investigation of temporal changes in the percentage of hair growth cycle stages in male and female mice Mice were humanely killed at two- or three-day intervals at the days indicated by blue triangles in Figure 2. Skin tissue specimens were collected from dorsal section S1 and used to histologically evaluate hair growth cycling. Dorsal skin specimens were fixed, embedded, sectioned at 5-μm thickness and exposed to HE and AP staining. Classification of hair growth cycle stages was based on histological location and morphology of each hair follicle, using a magnified view of the sections. (A and B, top) Results from HE staining in male (A) and female (B) specimens. Hair follicles were classified into the following four hair growth cycle stages: anagen I – IIIc, anagen IV – V, anagen VI – catagen VI and catagen VII – telogen. Numbers inside parentheses indicate total number of hair shafts successfully classified. (A, bottom) Representative sections are shown. Scale bars represent 500 μm. (B, bottom) Results from AP staining in female specimens. Hair follicles were classified into the following four hair growth cycle stages: anagen – catagen II, catagen III, catagen IV – catagen VIII and telogen. Fisher’s exact test was carried out on each day, (A and B, top) for a two-by-two contingency table (anagen I to V versus anagen VI to telogen, control versus LD reversal) and (B, bottom) for a two-by-two contingency table (anagen to catagen III versus catagen IV to telogen, control versus LD reversal). Asterisks beside each day in LD reversal represent a significant difference between the control and LD reversal groups on each day (∗*p* < 0.05, ∗∗*p* < 0.01).

Next, we histologically examined temporal changes in the percentage of hair growth cycle stages in male and female mice (Figure 3). After the hair growth cycle was synchronized among hair follicles by depilation of dorsal hair shafts, skin tissue specimens collected from dorsal section S1 at two- or three-day intervals were subjected to hematoxylin and eosin (HE) staining (Figures 3A and 3B, top). Although similar results would be obtained if other dorsal sections were used for histological evaluation, to increase the certainty of a clear comparison of hair cycling between the experimental groups, section S1, in which the effect of LD reversal was consistent from Days 12–18 in males, was selected and subjected to histological evaluation. Classification of hair growth cycle stage on the basis of the histological location and morphology of each hair follicle revealed that exposure of mice to LD reversal caused a delay in progression of the hair growth cycle, along with a prolonged anagen period. The statistical significance of this delay was tested using Fisher’s exact test on each day with a two-by-two contingency table (early to middle anagen versus late anagen to telogen, control versus LD reversal). In female mice, unlike males, the progression of hair cycling in LD-reversal mice unexpectedly appeared to accelerate at later hair cycle stages. This acceleration may be consistent with the lack of difference in hair shaft length between the groups on Day 18 (Figure 2C). In addition to HE staining, we confirmed this delay using alkaline phosphatase (AP) staining of skin tissue of female mice (Figure 3B, bottom). Although the role of AP in hair cycling remains undefined, it was previously reported that the location and strength of AP activity in hair follicles differ among hair cycle stages.^29^ We therefore utilized this activity as a marker to focus on progression from the catagen to telogen stages, which was relatively unclear in HE-stained specimens. Fisher’s exact test was carried out on each day with a two-by-two contingency table (catagen III and earlier versus catagen IV and later, control versus LD reversal). The data confirmed that the exposure of mice to LD reversal caused a delay in progression of the hair growth cycle. We evaluated the possibility of miniaturization by comparing the maximum width of hair follicles at similar hair stages in female mice, but found no statistical difference between the groups (Figure S4).

### Effects of repetitive LD reversal on autonomous shaft elongation *ex vivo*

**Figure 4:**
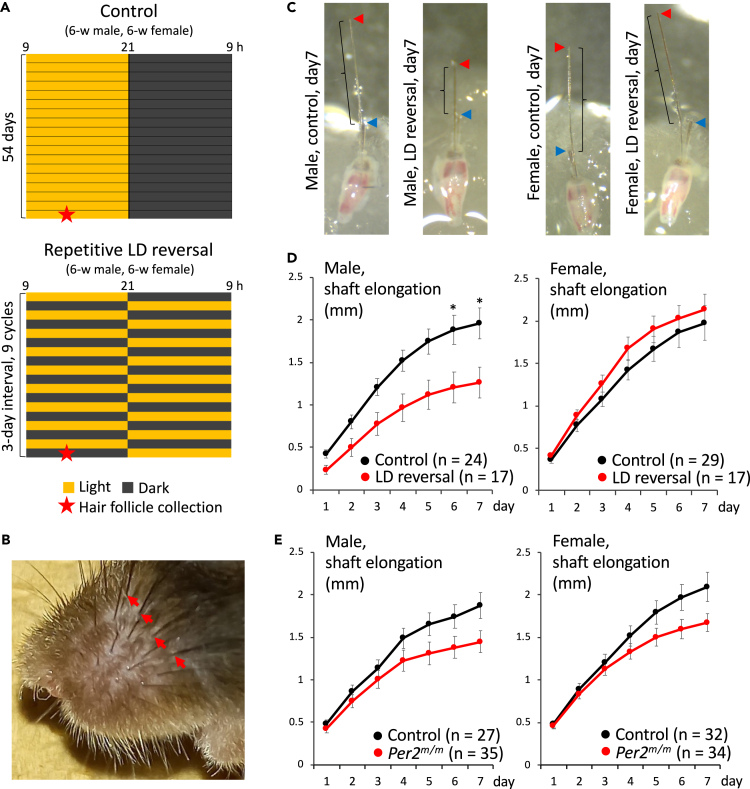
Effects of repetitive LD reversal on autonomous shaft elongation *ex vivo* (A) Schematic schedules of the autonomous shaft elongation assay using whisker hair follicles. After a regular 12:12 LD cycle for habituation, half of the six-week-old male and female wild-type C57BL6J mice were exposed to a repetitive LD reversal cycle at three-day intervals for a total of 54 days (B) An enlarged view of the mouse mystacial pad. To examine hair follicle-autonomous shaft elongation following exposure to the light environments, most caudal whisker hair follicles indicated by red arrowheads were surgically isolated at the day indicated by red stars in (a) and mid-anagen hair follicles were collected based on hair bulb morphology and shaft length. (C) Photographs show representative hair follicle-autonomous shaft elongation seven days after culturing. Mid-anagen hair follicles were isolated and cultured *ex vivo*. Red and blue arrowheads show the top of growing and mature hair shafts, respectively. Elongation of the growing hair shaft was quantified by measuring the distance between these tops. (D) The hair shaft of mid-anagen hair follicles was photographed and its elongation was quantified at 24-h intervals for a period of seven days. “n” and error bars represent the number of hair follicles examined and SEM, respectively. Results of a two-way mixed ANOVA (factor1, LD condition; factor2, day) were as follows: factor1, *p* < 0.01 (F = 7.59); factor2, *p* < 0.01 (F = 118); factor interaction, *p* < 0.01 (F = 4.87) in males, and factor1, *p* = 0.34 (F = 0.91); factor2, *p* < 0.01 (F = 139); factor interaction, *p* = 0.83 (F = 0.47) in females. Asterisks represent a significant difference in factor1 on each day (post hoc Tukey’s test, P∗ <0.05). (E) Six-week-old male and female wild-type and *Per2*^*m/m*^ mice were kept under a regular 12:12 LD cycle for a total of 54 days. Mid-anagen whisker hair follicles were surgically extracted and the hair shaft was grown *ex vivo*, whose elongation was quantified by comparing with the mature hair shaft. “n” and error bars represent the number of hair follicles examined and the SEM, respectively. Results of a two-way mixed ANOVA (factor1, genotype; factor2, day) were as follows: factor1, *p* = 0.11 (F = 2.68); factor2, *p* < 0.01 (F = 171); factor interaction, *p* < 0.01 (F = 4.34) in males, and factor1, *p* = 0.12 (F = 2.4); factor2, *p* < 0.01 (F = 204); factor interaction, *p* < 0.01 (F = 5.0) in females.

To evaluate the temporal dynamics of hair shaft growth in each individual hair follicle, we conducted *ex vivo* culture of mouse whisker hair follicles classified as mid anagen and examined their hair shaft elongation performance. Mouse whisker hair follicles are much larger than other hair follicles and therefore allow surgical isolation and *ex vivo* culture for the examination of hair follicle-autonomous shaft elongation. After male and female mice were exposed to repetitive LD reversal for 54 days, most caudal whisker hair follicles classified as mid anagen were isolated and cultured *ex vivo* on an absorbable gelatin sponge, and hair shaft length was measured for a period of seven days (Figures 4A and 4B). The results revealed that autonomous shaft elongation in mid anagen hair follicles was negatively affected by repetitive LD reversal only in male mice (Figures 4C and 4D), indicating a sex-dependent defect in hair follicle-autonomous shaft elongation caused by repetitive LD reversal.

Mice carrying a mutant *Period2* gene (*Per2*^*m/m*^ mice) showed normal body weight gain during the experimental period (Figure S1C), suggesting no severe health problems. The results of *ex vivo* culture of mid anagen hair follicles revealed that autonomous shaft elongation in both male and female *Per2*^*m/m*^ mice was likely less than in wild-type mice (Figure 4E). A two-way mixed ANOVA (factor1, genotype; factor2, hair shift length) showed no significant difference in factor1 in both sexes. However, the factor1-factor2 interaction was significant in both sexes, indicating that the genotype affected the temporal change in hair shaft growth. Given that the average hair shaft length was longer in *Per2*^*+/+*^ than *Per2*^*m/m*^ mice on almost all days and that the difference between the genotypes became wider with the passage of days, hair growth rate was estimated to be significantly faster in *Per2*^*+/+*^ than *Per2*^*m/m*^ mice in both sexes. Plots describing LD reversal-exposed versus *Per2*^*m/m*^ mice are shown in Figure S5. While a two-way mixed ANOVA (factor1, mouse treatment; factor2, hair shaft elongation) showed no significant difference in factor1 in both sexes, factor interaction was significant in females but not males. Given that the average was longer in LD reversal-exposed than *Per2*^*m/m*^ female mice on later days and that the difference became wider as days passed, hair growth rate was estimated to be significantly faster in LD reversal-exposed female mice.

## Discussion

While hair loss is not life-threatening, it does decrease quality of life, and potentially leads to psychological problems and the provocation of both anxiety and insecurity.^30^ Interest in hair loss is high around the world: IBISWorld, for example, a market research firm specializing in long-range forecasting, estimated that the hair loss treatment manufacturing industry in the United States would generate $4 billion in revenue in 2019. The category of hair loss most frequently encountered in primary care is nonscarring alopecia, which is further categorized as patterned, diffuse, or focal hair loss based on the distribution of loss on the scalp.^31^ The main cause of these three categories is a heritable polygenic trait, a wide range of inciting events, or an autoimmune disorder, respectively. Although no study has investigated the relation between circadian rhythm and hair loss in humans, animal studies using conventional gene knockout mice have demonstrated a negative impact of circadian dysfunction on hair growth. However, a study using conditional gene knockout mice indicated that this hair loss may reflect the loss of properties of clock protein that are independent of its role in the circadian clock. The drawing of conclusions on the effect of circadian dysfunction on hair growth therefore requires reevaluation using different experimental approaches. Gene knockout and knockdown differ fundamentally from circadian dysfunction in the real world, and may therefore provide only a minor advantage on translation to human health. In the present study, an aberrant light environment was experimentally generated by chronically exposing mice to repetitive LD reversal at three-day intervals, which caused forced and chronic circadian misalignment. Plasma concentration of testosterone is around ten times higher in male than in female mice.^32^ This difference in concentrations and/or plasma ratio of sex hormones has been reported to contribute to the sex-dependent difference in hair physiology and pathology in mice as well as in humans. For example, consistent with the finding that an increase in testosterone causes hair loss in women, injection of testosterone causes hair loss in female mice.^26^^,^^33^ In the present study, we therefore examined the sex-dependent response to repetitive LD reversal.

Although repetitive LD reversal could potentially cause unexpected stresses to mice, we concluded that the impaired hair growth we found in dorsal hair-sheared male mice under this reversal was mainly due to circadian dysfunction. Consistent with this result, synchronization of hair growth cycling following repetitive LD reversal by dorsal hair depilation revealed that hair regrowth impairment was likely attributable to a decrease in hair growth rate during anagen in both male and female mice. Histological investigation of the course of synchronized hair growth cycling indicates that a decrease in hair growth rate during anagen caused a prolongation of anagen period in the LD reversal group, probably because full hair regrowth required an extended anagen. Overall, although a previous study revealed that impaired hair growth in mice carrying a genetically dysfunctional circadian clock may be due to non-circadian dysfunction, the present study substantially contributes to defining a causal link between circadian dysfunction and hair growth impairment: our data, obtained from wild-type mice, demonstrate that chronic circadian misalignment - similar to that attributed to rotating shift work in a real world setting - causes impairment of hair growth in both males and females, probably due to a decrease in hair growth rate during anagen.

Under the hair-shearing conditions in Figure 1, our data show inconsistent results among sections; specifically, unlike sections S3 and S4, the LD reversal group showed a slightly but significantly longer average length of hair shafts than the control group in section S1, while both groups showed almost the same average length of hair shafts in section S2. These inconsistent results among sections are likely attributable to two causes: first, dorsal hair shafts were sheared during the second anagen after birth; and second, mouse hair cycling was more advanced in the rostral than caudal dorsal region. Specifically, while remaining hair shaft growth was more limited in the more rostral region due to hair-shearing later in anagen, remaining hair shaft growth was less affected in the more caudal region because hair-shearing was done earlier in anagen. Under these conditions, it was difficult to detect the difference in rostral hair growth between the two groups. Moreover, remaining hair shaft growth in the rostral region of control mice was further limited than that in LD-reversal mice because hair shaft growth was more advanced in controls than in LD-reversal mice. These factors might explain why hair shaft length was unexpectedly slightly longer in the LD reversal group than in the control group in section S1. The timing of hair-shearing in the present experiment is likely appropriate for evaluation of the effect of repetitive LD reversal on hair shaft growth in the caudal but not rostral regions.

Under the hair-depilation conditions in Figure 2, our data revealed minor sex-dependent differences in the effect of repetitive LD reversal on hair growth. In female mice, while the negative effect was detectable in earlier anagen (Day 12), when most hair shafts were immature and growing, it was undetectable in later anagen (Day 18) because most hair shafts grew nearly fully in both the control and LD reversal groups. Female hair shafts were longer than male ones in all dorsal sections on Day 18, indicating that female shafts grew and matured faster than male ones. It was reported that the anagen-to-catagen transition occurs around 18 days after dorsal depilation in C57BL6 females.^25^ On the other hand, in male mice, the data for hair shaft length on Day 18 clearly demonstrate that LD reversal caused a delay in hair growth. The difference in hair shaft length between the groups was detectable on this day because most hair shafts remained immature, unlike in female mice. However, a convincing interpretation of the results on Day 12 is difficult, because the data vary too widely among not only the dorsal sections but also the hair shafts in each section. This inconsistent and unnatural data on Day 12 might not reflect the true situation, due to the following technical issues. Figure 2B shows that male hair shaft length varied very largely on Day 12. In this case, very short hair shafts were hard to grasp with tweezers; in particular, hair shafts of less than 1 mm likely underwent negative selection. Also, after being plucked out, it was hard to find these very short shafts because they likely lay hidden in the shadows and behind long hair shafts. Figure 2B indicates that these issues were likely to occur in male samples on Day 12. Indeed, hair shafts of less than 1 mm seem to be unnaturally lost in sections S2 and S4 in the LD reversal group, which might explain why average hair shaft length was unexpectedly longer in the LD reversal than control groups.

We also evaluated the temporal dynamics of hair shaft growth in each individual hair follicle *ex vivo* and successfully confirmed that LD reversal caused a delay in hair growth*,* by examining autonomous shaft elongation of isolated and cultured whisker hair follicles classified as mid anagen. Unlike dorsal hair shafts under hair-depilation conditions, we detected an impairment in male but not female whisker shaft growth, potentially suggesting that the sex-dependent effects of chronic LD reversal on shaft elongation may differ among types of hair. However, the results from the *in vivo* dorsal hair regrowth assay under a depilation condition indicate that a hair growth delay in females occurred in earlier stages of anagen than in males. Given that hair follicles in later anagen, designated here as mid anagen, were used for the *ex vivo* whisker shaft elongation assay, the lack of difference in *ex vivo* hair growth between the female groups supports the hypothesis that LD reversal likely affected earlier but not later anagen in female mice. Although a similar impairment might be detectable in female mice, as in male mice, if earlier anagen hair follicles were used for this *ex vivo* assay, our *ex vivo* culture method is difficult to apply to whisker hair follicles in earlier anagen. Given that there was no difference in female dorsal hair length on Day 18 in Figure 2C, hair shaft growth in the LD reversal groups might have been more active than that in the control groups in later anagen, which may in turn be consistent with the slightly – albeit not significantly – faster *ex vivo* hair growth in the LD reversal group than in the control group in Figure 4D. In the case of male mice, in contrast, the LD-reversal-induced delay in dorsal hair growth during later anagen on Day 18 in Figure 2C is consistent with the delay in *ex vivo* hair shaft growth in Figure 4D.

Autonomous shaft elongation was also likely delayed in *ex vivo*-cultured mid-anagen whisker hair follicles isolated from *Per2*^*m/m*^ mice kept under normal LD cycles, indirectly suggesting that the chronic LD reversal-delayed hair shaft elongation was due to dysfunction of autonomous circadian clocks within the hair follicle cells. As the circadian clock reportedly plays a role in cell cycle gating in hair follicle tissue,^19^^,^^34^ circadian dysfunction may result in aberrant cell proliferation, which may in turn lead to impairment of hair growth. Autonomous shaft elongation was unexpectedly delayed *ex vivo* in not only male but also female *Per2*^*m/m*^ mice, which is inconsistent with the sex-dependent effect of repetitive LD reversal on *ex vivo* shaft elongation in wild-type mice. However, this discrepancy may be explained by the hypothesis that whereas *ex vivo* shaft elongation in mid-anagen hair follicles was not affected in LD reversal-treated females because this light condition was effective only during earlier anagen, as described above, genetic circadian dysfunction continued to be effective on *ex vivo* shaft elongation from earlier to later anagen. In other words, chronic LD reversal alone was not sufficient for continuous impairment of hair shaft growth up to later anagen in females, as it did not cause as severe and irreversible circadian dysfunction within hair follicles as that in mice carrying a dysfunctional clock gene. Androgenic hormones, with much higher concentrations in males than females, might be an additional factor in impairment of hair shaft growth during later anagen. However, we cannot exclude the possibility that, as shown in *Bmal1* knockout mice in previous studies and mentioned in the Introduction, the different result from those in repetitive LD-reversal experiments using wild-type mice indicates hair growth impairment in *Per2*^*m/m*^ mice is caused by non-circadian dysfunction.

In summary, the present study demonstrates that circadian misalignment causes a decrease in hair growth rate during anagen in both sexes, albeit with minor sex-dependent differences. Long-term circadian misalignment such as in chronic social jet lag and rotating shift work is therefore a potential risk factor for human hair loss. Improvement in the light environment or amelioration of circadian dysfunction may therefore be a promising approach to delaying the development and progression of hair loss in both males and females.

### Limitations of the study

There are a few limitations in this study which warrant attention. First, our results may not be directly applicable to hair loss in humans at this time because of several differences between mice and humans. More specifically, there are two major issues in applying our results to humans: mice are nocturnal, unlike humans, and hair physiology differs between mice and humans.^35^^,^^36^ While the former can be solved simply by performing similar experiments using a diurnal animal as a model, no animal model might be able to solve the latter. Rather, overcoming the latter issue would require experiments using human tissue or bodies. However, applying our present experimental protocol to humans would be nearly impossible, because monitoring human scalp hair cycling takes several years. Given this limitation, it is realistic to use epidemiological approaches targeting subjects engaged in rotating shift work over many years. For example, statistical comparison of hair density between day and rotating shift workers using multivariate analyses which take account of the difference in age, sex, business type, and working schedule among subjects may reveal the correlation between circadian misalignment and hair loss. Considering these issues together, the drawing of solid conclusions on whether chronic circadian misalignment accelerates hair loss in humans therefore awaits epidemiological studies on the association between rotating shift work or social jet lag and hair loss. Second, our study does not provide conclusive evidence that the negative effect of repetitive LD reversal on hair growth occurs really through circadian dysfunction. A recent study indicated that external blue light input via the retina activates hair growth through a sympathetic neural pathway.^37^ Given the well-known finding that aberrant light environments cause autonomic dysregulation,^38^^,^^39^^,^^40^^,^^41^ it is feasible that functional impairment in a sympathetic neural pathway causes a delay in hair growth in a circadian-independent manner.

## Resource availability

### Lead contact

Further information and requests for resources and reagents should be directed to and will be fulfilled by the lead contact, Makoto Akashi (akashima@yamaguchi-u.ac.jp).

### Materials availability

This study did not generate new unique reagents.

### Data and code availability

• The datasets generated and analyzed during the current study are available from the lead contact on reasonable request.
• This paper does not report the original code.
• Any additional information required to reanalyze the data reported in this paper is available from the lead contact upon request.

## Acknowledgments

We thank Nanami Yasumune, Yuna Yamamoto, Junko Sumino and Ritsuko Matsumura for their expert technical assistance. We express our great appreciation to Yutaka Shimomura (Yamaguchi University), Riuko Ohashi (Niigata University), Tomoharu Sato (Osaka University) and Takashi Matsuzaki (Shimane University) for technical advice.

## Author contributions

M.A. conceived and supervised the project and wrote the manuscript. Y.M., A.N., K.F., A.M., T.H., Y.M., M.N., and Y.S. performed experiments. I.T.T. analyzed data. K.N. provided general supports and gave conceptual advice.

## Declaration of interests

The authors declare no competing financial interests.

## STAR★Methods

### Key resources table

**Table:** 

REAGENT or RESOURCE	SOURCE	IDENTIFIER
**Chemicals, peptides, and recombinant proteins**		

Dulbecco’s Modified Eagle’s Medium	Nacalai Tesque	# 08456
Fetal bovine serum	Sigma-Aldrich	# 173012
Penicillin and Streptomycin	GIBCO	# 15070063
Gelfoam	Pfizer	# 7028247
Williams E medium	Sigma-Aldrich	# A12176
GlutaMax	Thermo Fisher Scientific	# 35050
Insulin	Eli Lilly	N/A
Hydrocortisone	Sigma-Aldrich	# H0888

**Critical commercial assays**		

NBT/BCIP Stock Solution	Roche	# 11681451001

**Experimental models: Organisms/strains**		

C57BL/6JmsSlc mice	Japan SLC	N/A
*Per2*^*m/m*^ mice	Jackson Laboratories	Stock number 003819

**Software and algorithms**		

Clock Lab software	Actimetrics Inc	N/A
3R Anyty software	3R SOLUTION Corp	N/A

**Other**		

iButton, Thermochron Type-G	KN Laboratories	N/A
Plastic tool dip	Plasti Dip International	N/A
Wired microscope	3R SOLUTION Corp	N/A

### Experimental model and study participant details

#### Animals

Five-week-old male and female wild-type C57BL6J mice were purchased from Japan SLC. *Per2*^*m/m*^ mice (stock number 003819) were purchased from Jackson Laboratories and then repetitively backcrossed with black-haired wild-type C57BL6J mice. In the original strain of *Per2*^*m/m*^ mice, pigment is completely absent from skin, hair and eyes because they are homozygous for *Tyr*, making it difficult to visually identify and surgically isolate hair follicles. Genotyping of *Per2*^*m/m*^ mice was performed with PCR according to the supplier's instructions. Mice were bred and maintained on a regular 12:12 light-dark (LD) cycle (lights on at 9:00 A.M.) and allowed *ad libitum* access to food and water. To perform experimental exposure of mice to a chronic aberrant light environment, each six-week-old wild-type mouse was housed singly in a standard mouse cage (W 213 mm, D 324 mm and H 131 mm) equipped with a sensor to record locomotor activity. Access to food and water was provided *ad libitum*. For habituation, the mice were maintained on a regular 12:12 LD cycle during the first seven days of activity recording. Then, mice aged around six to seven weeks after birth, when their dorsal hair cycle stage corresponds to around the first catagen after birth, were exposed to repetitive reversal of LD cycle at three-day intervals for a total of 54 days. Locomotor activity was measured using an infrared thermal sensor and recorded using Clock Lab software (Actimetrics Inc.). To perform core body temperature recording, mice were anesthetized with isoflurane gas and an iButton (Thermochron Type-G, KN Laboratories) was surgically implanted in the abdominal cavity. We programmed the iButtons to record the temperature every 90 min and waterproofed them with clear plastic tool dip (Plasti Dip International) before implantation. Body weight was measured right before and after repetitive LD reversal. All protocols for animal experiments were approved by the Animal Research Committee of Yamaguchi University. Animal studies were performed in compliance with the Yamaguchi University Animal Care and Use guidelines.

### Method details

#### *In vivo* dorsal hair growth assay

*In vivo* dorsal hair growth was investigated under two different experimental conditions: hair-shearing and hair-depilation.

To perform the assay in the former condition, immediately after repetitive LD reversal for 54 days (day 0), mice were anesthetized with isoflurane gas and their dorsal hair shafts were neatly sheared using grooming clippers, to an average hair shaft length of approximately 0.7 mm. After recovering from anesthesia, they were returned to the same experimental condition. 18 days after hair shearing, the dorsal area from the root of the neck to the base of the tail was divided into four equal sections using a grid frame, and then a few hundred hair shafts were randomly collected from each section simply by plucking them using tweezers, and the length of each individual hair shaft was measured under a stereo-microscope. More specifically, to compare quantitative and statistical difference in hair growth between experimental groups, dorsal hair shafts were photographed with a wired small microscope (3R SOLUTION Corp., Japan). The length of each plucked hair shaft was measured based on digital images with the 3R Anyty software (3R SOLUTION Corp., Japan).

To perform the assay in the second condition, following repetitive LD reversal for 54 days, mice were anesthetized with isoflurane gas, and their dorsal hair shafts were sheared and then completely removed by waxing. As described for the first condition, growing dorsal hair shafts were plucked using tweezers under unanesthetized conditions. Because depilation of mouse hair shafts induces anagen entry and consequently synchronizes hair growth cycling among hair follicles,^25^ relative to the first condition this procedure facilitates *in vivo* evaluation of the effect of chronic repetitive LD reversal on hair shaft elongation or growth cycling. However, a slight inflammation caused by waxing-induced wounding has been demonstrated^42^ and may potentially affect experimental results.

#### Histological evaluation of hair growth cycle

After LD reversal for 54 days, hair growth cycle was synchronized among hair follicles by depilation of dorsal hair shafts using wax. Mice were humanely killed using isoflurane at two- or three-day intervals and skin tissue specimens were collected and used to visually and quantitatively evaluate hair growth cycling. Briefly, dorsal skin specimens were fixed in 4% paraformaldehyde, dehydrated with increasing concentrations of ethanol, cleared with xylene and embedded with paraffin. Paraffin blocks were sectioned at 5 μm using a rotary microtome. Sections were placed on a slide glass and deparaffinized first with xylene and then sequentially decreasing concentrations of ethanol. Hematoxylin & eosin (HE) and alkaline phosphatase (AP) staining were performed according to a standard method and the manufacturer’s instructions (NBT/BCIP Stock Solution, Roche), respectively. After staining, the sections were dehydrated and enclosed with Canada balsam under cover glasses. A magnified view of the sections was photographed using a conventional optical microscope, and stored digital images were used to identify hair growth cycle stages. According to previous reports,^25^^,^^29^^,^^43^^,^^44^ on the basis of hair bulb location, morphology and shaft length, hair follicles were classified into the following hair growth cycle stages: for HE staining, anagen I – IIIc, anagen IV – V, anagen VI – catagen VI and catagen VII – telogen; for AP staining, anagen – catagen II, catagen III, catagen IV – catagen VIII and telogen.

#### Surgical isolation of mouse whisker hair follicles

Mouse whisker hair follicles were isolated by microdissection using a previously described method with a minor modification.^45^ Briefly, after euthanasia, both the left and right mystacial pads were surgically removed from the mice, washed vigorously two times with 70% ethanol and three times with phosphate-buffered saline (PBS), and then placed in Dulbecco's modified Eagle's medium (DMEM, Nacalai, Japan) supplemented with 1% penicillin and streptomycin. Individual whisker hair follicles were carefully dissected under a dissecting microscope. Considerable care was taken to remove surrounding connective tissue but not to damage hair follicles. Whisker hair follicles were collected and transferred to a Petri dish containing fresh DMEM. Hair growth cycle stages of the whisker hair follicles were visually classified under a stereoscopic microscope according to criteria described below. In accordance with previous reports with minor modifications,^43^^,^^44^ on the basis of hair bulb morphology and shaft length, isolated whisker hair follicles were classified into hair growth cycle stages, namely pro-anagen (PA), early anagen (EA), mid anagen (MA) and late anagen (LA). In mouse whisker hair follicles, termination of hair growth in the previous hair cycle and initiation of hair regeneration in the subsequent cycle partially overlap, and telogen is therefore not obvious. EA-, MA- and LA-follicles were categorized simply based on the relative length of the growing new hair shaft to the fully grown old one, namely EA, less than one-quarter; MA, between one-quarter and two-thirds; and LA, over two-thirds. Hair follicles whose shaft was not yet produced or already produced but under the skin surface were classified as PA. Catagen follicles have only one hair shaft because the old one has already shed at the beginning of early catagen.

#### *Ex vivo* culture of whisker hair follicles

To examine the performance of hair follicle-autonomous shaft elongation, after exposure of mice to repetitive reversal of LD cycle at three-day intervals for a total of 54 days, whisker hair follicles were isolated, classified and cultured *ex vivo*. Briefly, hair follicles in mid anagen were collected by surgical isolation and morphologically classified as described above. To facilitate the measurement and quantification of hair shaft elongation in anagen hair follicles, both the matured and growing new hair shafts were cut into the same length with a surgical knife, and hair follicles were then singly placed on an absorbable gelatin sponge (Gelfoam, Pfizer Japan Inc.) located inside individual wells of 24-well multi well plates. The hair shaft of hair follicles was grown in 500 μl of Williams E medium (Sigma-Aldrich, Japan) supplemented with 2 mM GlutaMax (Thermo Fisher Scientific, USA), 10 μg insulin per mL (Eli Lilly, Japan), 10 ng hydrocortisone per mL (Sigma-Aldrich, Japan) and 1% penicillin/streptomycin (Thermo Fisher Scientific, USA) at 36.5°C in an atmosphere of 5% CO_2_. To record digital images of hair follicles using a wired small microscope (3R SOLUTION Corp., Japan), culture plates were taken out of a CO_2_ incubator and photographed at 24-h intervals for a period of seven days. The elongation of the growing hair shaft was measured and quantified by comparison with the matured hair shaft using stored digital images with the 3R Anyty software (3R SOLUTION Corp., Japan).

### Quantification and statistical analysis

In general, data are represented by mean ± SD. Specific statistical tests used, p value level definitions, and additional details are listed in figure legends.
