# Supplementary material for: Chronic circadian misalignment is a risk factor for hair growth impairment
Source: iScience. 2024 Sep 18;27(10):110974. doi: 10.1016/j.isci.2024.110974 (PMC11615142; doi:10.1016/j.isci.2024.110974)
Supplement: Document S1. Figures S1–S5 [file mmc1.pdf]

iScience, Volume *27*

Supplemental information

Chronic circadian misalignment is a risk factor for hair growth impairment

Yoshiki Miyawaki, Atsuhiro Nishida, Keisuke Fukushima, Aoi Matsumoto, Teruki Hamano, Yukiya Mori, Mamoru Nagano, Isao T. Tokuda, Yasufumi Shigeyoshi, Koichi Node, and Makoto Akashi

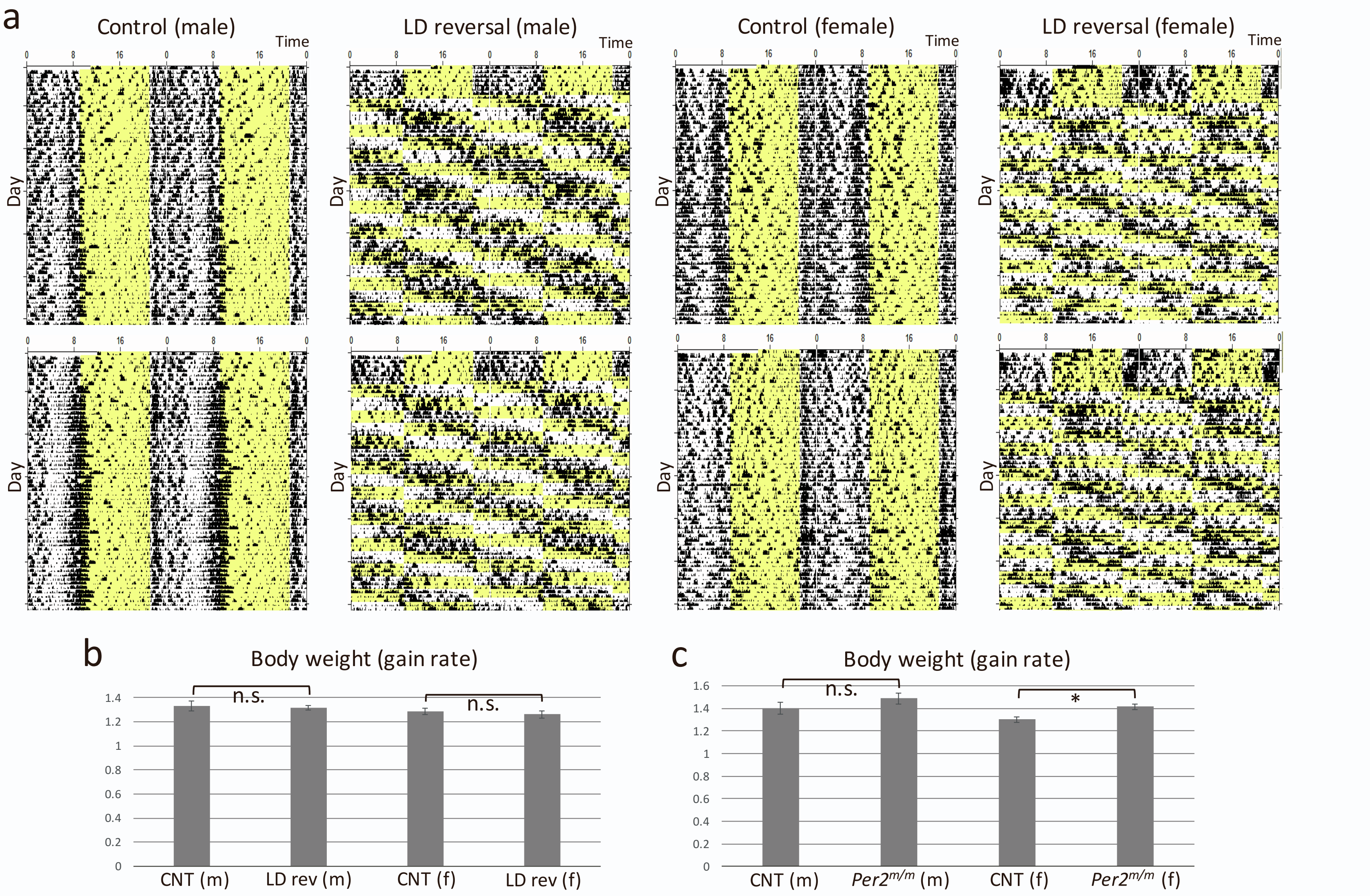

Figure S1

Effects of repetitive LD reversal on locomotor activity and body weight (Related to Figure 1)

(a) Six-week-old male and female wild-type C57BL6J mice were housed singly. After a regular LD cycle for habituation, the mice were exposed to a regular or repetitively reversed LD cycle for a total of 54 days. Locomotor activity was monitored in real time with infrared sensors. Two representative actograms are shown for each experimental group. The yellow area indicates the light period. (b) Body weight of male (m) and female (f) wild-type C57BL6J mice was measured immediately before and after the 54-day regular LD (CNT) or 54-day repetitively reversed LD (LDrev) cycle. (c) Body weight of male (m) and female (f) wild-type and *Per2*$^{m/m}$ mice was measured immediately before and after the 54-day regular LD cycle. (b and c) The rate of body weight gain was calculated as the fold change in body weight. Error bars represent the SEM. An unpaired t-test was performed between the indicated experimental groups (*P < 0.05). n.s. represents no significant difference.

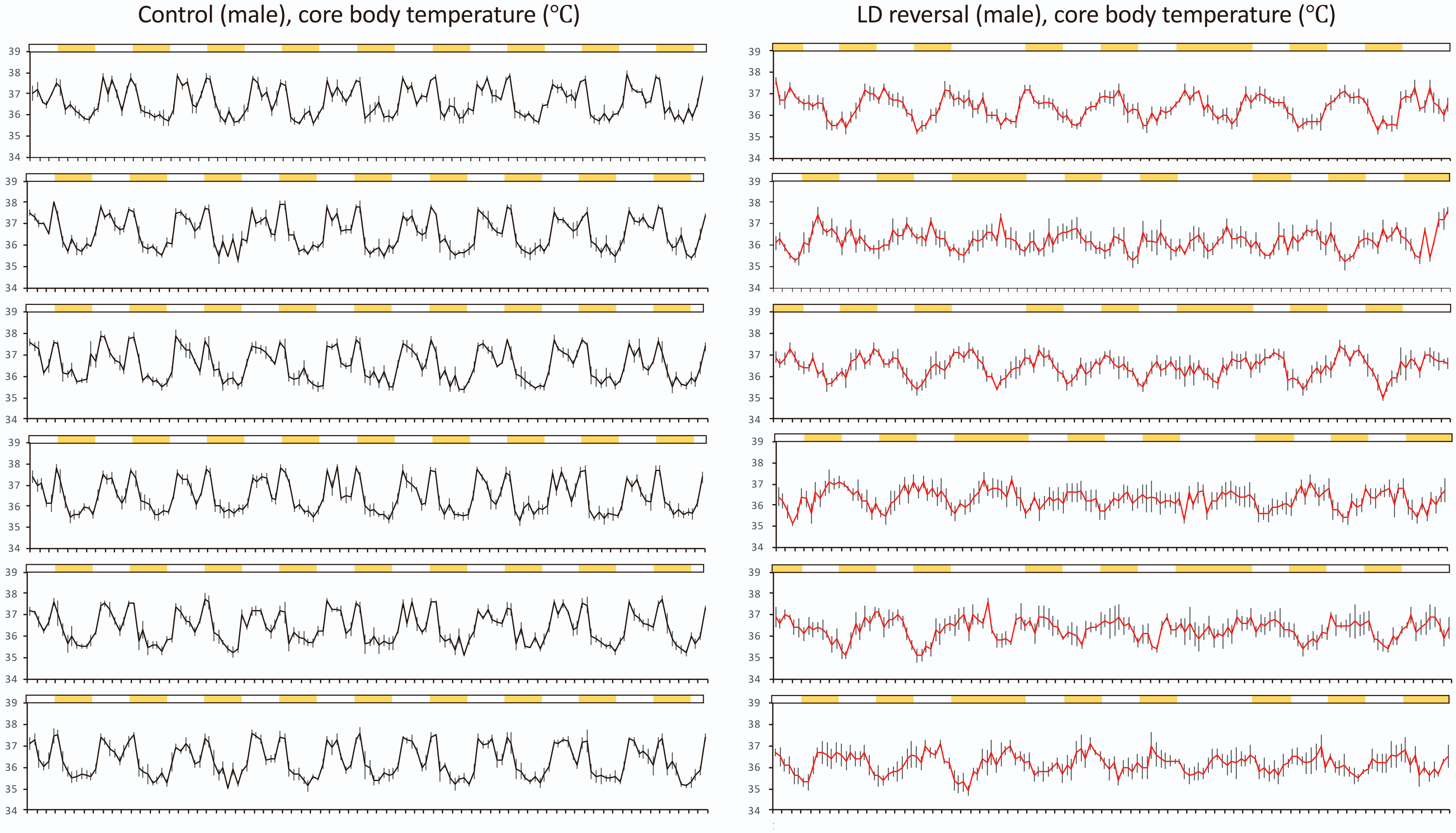

Figure S2

Effect of repetitive LD reversal on circadian rhythms in core body temperature (related to Figure 1)

Six-week-old male wild-type C57BL6J mice were housed singly. After a regular 12-h light and 12-h dark cycle for habituation, the mice were exposed to a continued regular or repetitively reversed LD cycle for a total of 54 days. Core body temperature was monitored using iButtons. The average core body temperature of five mice with ± SE is shown for each experimental group. The yellow areas indicate light period.

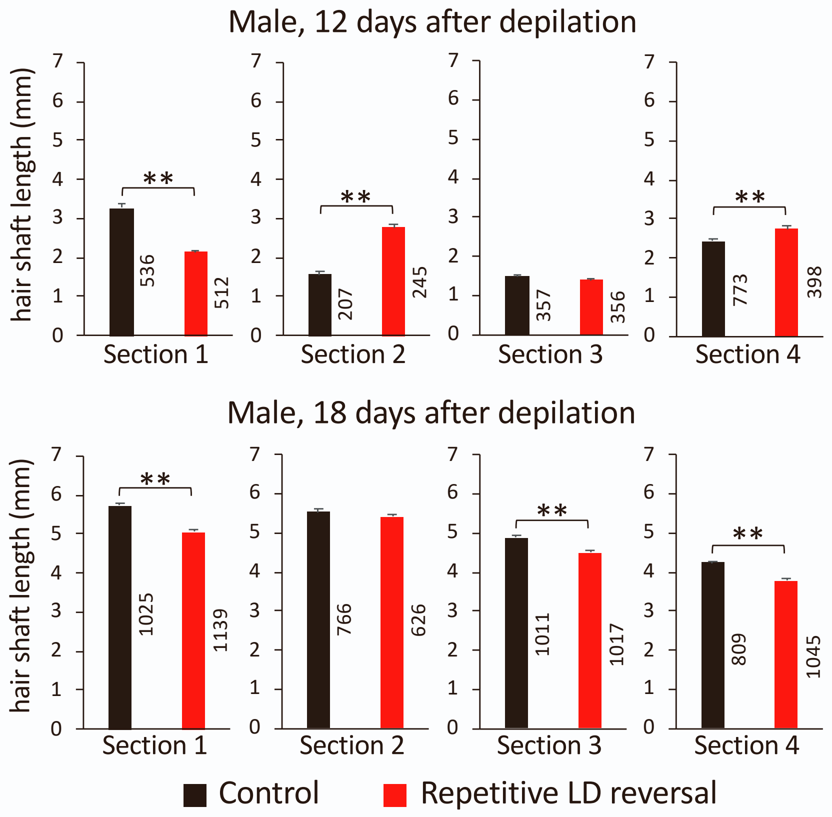

Figure S3

Reevaluation of the result shown in Figure 2c by excluding hair shafts potentially collected from desynchronized dorsal area (related to Figure 2)

Statistical reevaluation of the result in Figure 2c used data without hair shafts collected from dorsal sections that included any patch. Numbers beside columns and error bars indicate the number of hair shafts examined and the standard error of the mean (SEM), respectively. A two-way ANOVA with post hoc Tukey's test (factor1, LD condition; factor2, section) was performed on each day. Asterisks represent a significant difference between LD conditions in each section (*$p < 0.05$, **$p < 0.01$).

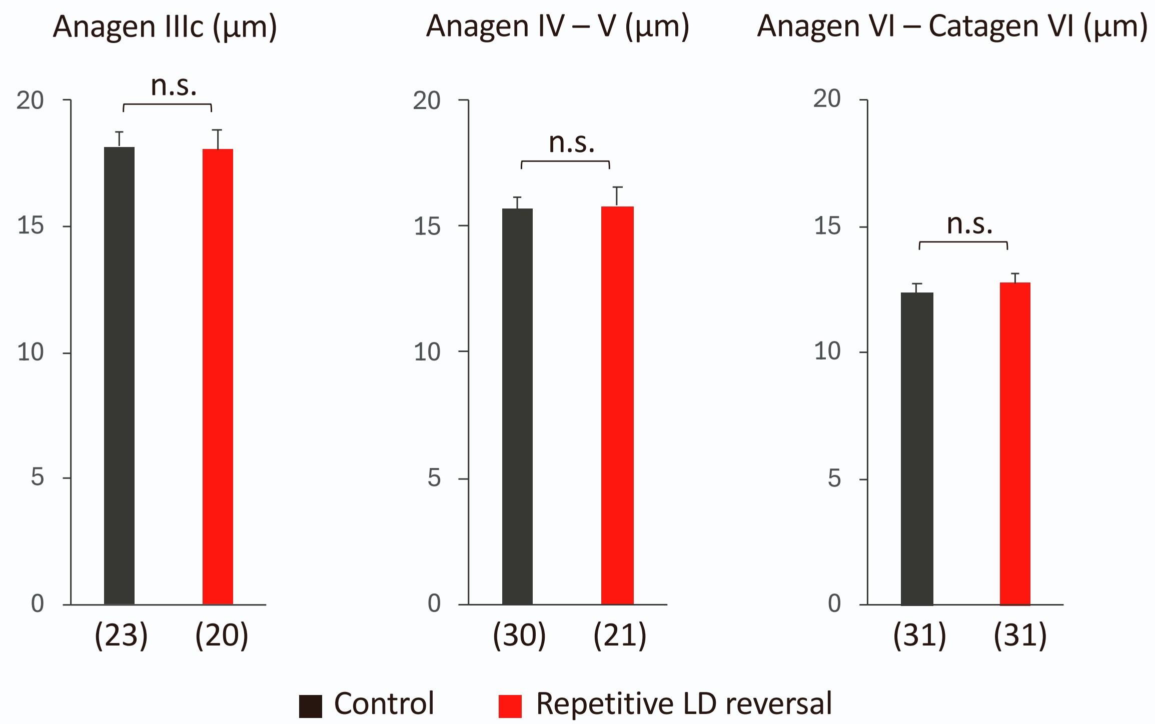

Figure S4

Effect of repetitive LD reversal on hair follicle size in female mice (related to Figure 3)

Average maximum width of hair follicles at each hair cycle stage. Maximum width of each hair follicle was measured using the image J software. Numbers under columns indicate the number of hair follicles examined and error bars represent the SEM. An unpaired t-test was performed between the indicated experimental groups. n.s. represents no significant difference.

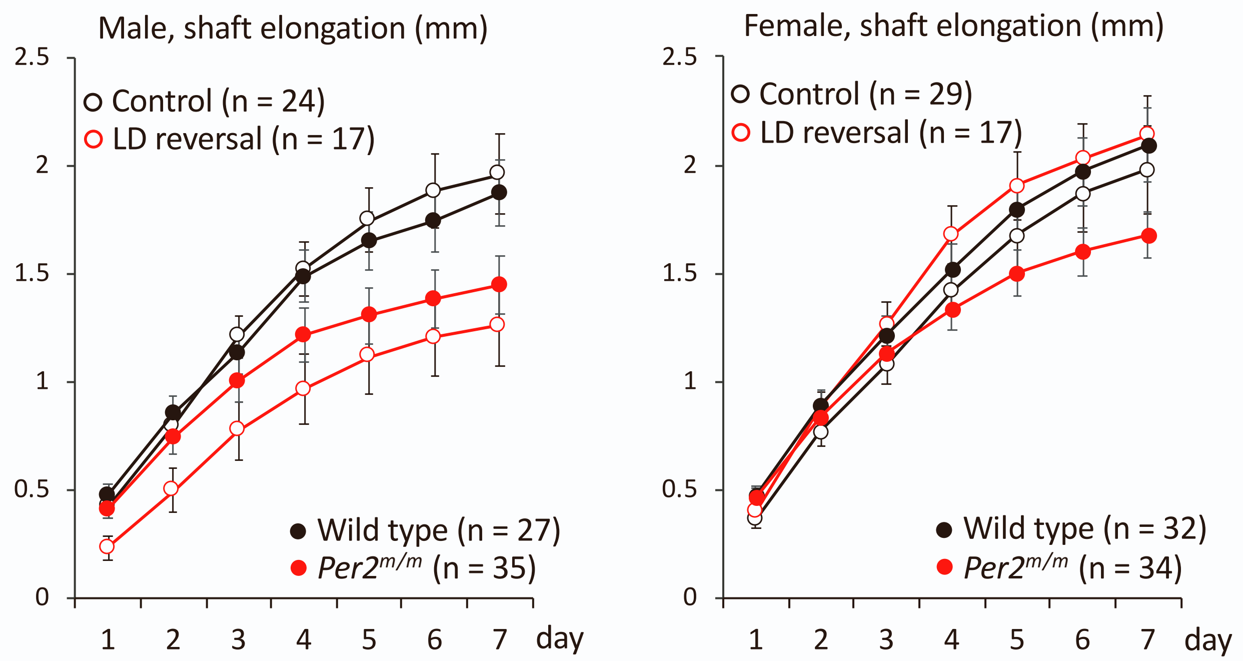

Figure S5

Effect of repetitive LD reversal and *Per2* mutation on *ex vivo* shaft elongation (related to Figures 4d and 4e)

Plots shown in Figures 4d and 4e were combined to visually and statistically compare the difference in *ex vivo* shaft elongation between the LD reversal (open red circle) and *Per2*$^{m/m}$ (filled red circle) groups. Results of a two-way mixed ANOVA (factor1, LD reversal and Per2$^{m/m}$; factor2, day) were as follows: factor1, $p = 0.24$ ($F = 1.42$); factor2, $P < 0.01$ ($F = 105$); and factor interaction, $p = 0.98$ ($F = 0.20$) in males, and factor1, $p = 0.075$ ($F = 3.3$); factor2, $P < 0.01$ ($F = 222$); and factor interaction, $P < 0.01$ ($F = 8.2$) in females.
